# Assessment of Burden Among Family Caregivers of Schizophrenia: Psychometric Testing for Short-Form Zarit Burden Interviews

**DOI:** 10.3389/fpsyg.2018.02539

**Published:** 2018-12-19

**Authors:** Yu Yu, Zi-wei Liu, Wei Zhou, Xiao-chuan Chen, Xing-yu Zhang, Mi Hu, Shui-yuan Xiao

**Affiliations:** ^1^Xiangya School of Public Health, Central South University, Changsha, China; ^2^Mental Health Institute of the Second Xiangya Hospital, National Clinical Research Center on Mental Disorders & National Technology Institute on Mental Disorders, Hunan Key Laboratory of Psychiatry and Mental Health, Central South University, Changsha, China; ^3^Xiangya Hospital, Central South University, Changsha, China; ^4^Traditional Chinese Medical Hospital of Ya'an, Sichuan, China

**Keywords:** Zarit Burden Interview (ZBI), short-forms, psychometric testing, reliability, validity

## Abstract

**Objective:** Although various short forms of Zarit Burden Interview (ZBI) have been developed, there is a lack of standard psychometric testing and comparison among them. The study aims to examine the psychometric properties of ten short versions of the most frequently used ZBI among a sample of schizophrenia caregivers and to find the one with the best performance.

**Methods:** Cross-sectional door-to-door survey of ZBI-22 and a series of validated instrument data from 327 family caregivers of schizophrenia patients in a Chinese rural community were conducted from October 2015 to January 2016. Reliability was assessed using McDonald's omega coefficient (ω). Validity including concurrent validity, known group's validity, and criterion validity were assessed by Spearman correlations and Mann-Whitney U tests. Overall discrimination ability was evaluated using the area under the receiver operating characteristic curve (AUC).

**Results:** Reliability was generally good for all short forms (ω = 0.69–0.84), except for the Gort ZBI-4 (ω = 0.58), which is acceptable considering its small item numbers. Concurrent validity was good across all various ZBI forms with significant negative correlations with patient's function (*r* = −0.34 to −0.48, *p* < 0.01), as well as significant positive correlations with caregiver's depression (*r* = 0.49–0.65, *p* < 0.01), and anxiety symptoms (*r* = 0.45–0.58, *p* < 0.01). Known groups' validity (carers with disease vs. without disease; carers being parents vs. spouse vs. others) showed inconsistent results among various short forms. Criterion validity was generally good for all short forms with significant positive correlations with Family Burden Interview Schedule (*r* = 0.67–0.75, *p* < 0.01), except for the Higginson ZBI-1(*r* = 0.57, *p* < 0.01). Discriminative ability was also good for all short forms (AUC range: 0.85–0.99), with various cutpoints proposed. Among all ten short forms, the Ballesteros ZBI-12 and the Gort ZBI-7 outperformed others with almost equally good performance in comprehensive psychometric testing.

**Conclusions:** This study provides support for the reliability, validity, and discriminative ability of the ten various short forms of ZBI for use among schizophrenia family caregivers, with the Ballesteros ZBI-12 and the Gort ZBI-7 endorsed as the best ones.

## Introduction

The Zarit Burden Interview (ZBI) is one of the oldest and most commonly used instruments for assessing caregiving burden at an international level (Knight et al., 2000). Initially developed more than 30 years ago, the ZBI was intended to measure burden and stress experienced by caregivers caring for people with dementia with 29 items on a four-point Likert type scale (Zarit et al., 1980). A revised ZBI (Zarit et al., 1985a,b) was later introduced with 22 items on a five-point Likert type scale, which has been widely translated into various languages, and validated across countries and cultures, such as Europe (Braun et al., 2010; Martin-Carrasco et al., 2010; Chattat et al., 2011), Africa (Imarhiagbe et al., 2017) and Asian countries like China (Wang et al., 2008; Lu et al., 2009) and Japan (Hirono et al., 1998) allowing for international comparison. Evidence also shows that the ZBI can be interpreted similarly across gender or across educational level (Lin et al., 2017a). Furthermore, a meta-analytic study has proved that the ZBI are reliable across populations of caregivers (i.e., spouses/partners, children, and parents), care-recipients (i.e., AD/dementia, physical illness, and mental illness), and most language versions (e.g., French, Spanish, and Chinese) (Bachner and O'Rourke, 2007).

The widespread use of ZBI in both research and clinical settings worldwide has fostered interest in simplification of the 22-item scale for ease of administration, minimization of respondent burden, and quick screening in busy occasions (Lin et al., 2017b). A number of short forms of ZBI have been proposed, with the number of items ranging from 1 to 14, as shown in Table 1. All those short forms are developed or assessed either by using classical test theory or item response theory, with theory-guided choice of item selection. Comparison of various abridged versions of ZBI have been conducted with consistent findings supporting for the reliability and validity of various short forms, while uniformly endorsing as the best one either the Bedard 12-item ZBI (Higginson et al., 2010; Hagell et al., 2017) or the Ballesteros 12-item ZBI (Lin et al., 2017b).

**Table 1:** Items included in the short forms of the Zarit Burden Interview^*^.

**No**.	**ZBI-22^**&**^ Items Content (Abridged)**	**Higginson et al., 2010, ZBI-1, 3 disease^**#**^**	**Bedard et al., 2001, ZBI-4 dementia**	**Gort et al., 2010, ZBI-4 dementia**	**Higginson et al., 2010, ZBI-6, 3 disease^**#**^**	**Gort et al., 2005, ZBI-7, palliative care**	**Zhou**, 2011, **ZBI-7 chronic dis^$^**	**Arai et al., 2003, ZBI-8 dementia**	**Bedard et al., 2001, ZBI-12 dementia**	**Ballesteros et al., 2012, ZBI-12 dementia**	**Knight et al., 2000, ZBI-14 dementia**
1	Relative asks for more help than needed										
2	Not enough time for yourself		X	X	X	X	X		X	X	X
3	Stressed between caring and other responsibilities		X		X	X	X		X	X	
4	Embarrassed over behaviors						X	X			X
5	Angry when around your relative			X				X	X		X
6	Relative affects your relationship with others				X	X		X	X		X
7	Afraid of what the future holds for relative										
8	Your relative is dependent on you									X	X
9	Strained when around your relative		X	X	X	X		X	X	X	X
10	Your health has suffered because of caring				X	X	X		X	X	X
11	Insufficient privacy because of your relative						X		X	X	X
12	Social life has suffered because of caring							X	X	X	X
13	Uncomfortable having friends over because of relative							X			X
14	Relative seems to expect you to take care of him or her, as if you were the only one to depend on			X							X
15	Do not have enough money to care for your relative										
16	Not able to take care of your relative much longer									X	
17	Lost control of your life since your relative's illness				X	X	X		X	X	
18	Wish you could leave the care to someone else							X		X	X
19	Uncertain about what to do about relative		X			X	X	X	X		
20	You should be doing more for your relative								X		X
21	Could do a better job caring for your relative								X		X
22	Overall, how burdened do you feel in caring	X				X				X	
	McDonald's omega coefficient		0.69	0.58	0.77	0.80	0.84	0.84	0.84	0.85	0.83
	Correlations with the ZBI-22^@^	0.63	0.85	0.83	0.91	0.92	0.92	0.91	0.96	0.96	0.96

* *Item response categories: 0 = never, 1 = rarely, 2 = sometimes, 3 = quite frequently, 4 = nearly always (except Item 22: 0 = not at all, 1 = a little, 2 = moderately, 3 = quite a bit, and 4 = extremely)*.

*Total score ranges (higher = more burden): ZBI-1 = 0–4; ZBI-4 = 0–16; ZBI-6 = 0–24; ZBI-7 = 0–28; ZBI-8 = 0–32; ZBI-12 = 0–48; and ZBI-14 = 0–56*.

& *ZBI-22 = 22-Item Zarit Burden Interview*.

# *Advanced cancer, Dementia, Acquired brain injury*.

$ *Mental illness, Rheumatoid arthritis(RA), Cancer, Cerebral stroke, Chronic obstructive pulmonary disease (COPD), Dabietes, Coronary Heart Diseas(CHD)*.

@ *Coefficients by spearman test*.

However, these previous performance comparisons fall short in four key aspects: First, among all ten short forms reported in the literature, only six versions have been assessed, rendering the comparison incomplete, and inconclusive (Higginson et al., 2010; Hagell et al., 2017; Lin et al., 2017b). Secondly, the majority of short forms were developed and tested in a population of dementia caregivers, little evidence exists on their psychometric properties among caregivers of mental illness, although the full 22-item form has already been widely used, and validated among such a population (Higginson et al., 2010; Flynn Longmire and Knight, 2011; Tang et al., 2016; Hagell et al., 2017; Lin et al., 2017b). Thirdly, previous validity tests were based on comparison between short forms of ZBI with self-designed questions, such as informal care hours and financial situations (Lin et al., 2017b), or using the full 22-item version as a self-comparison (Higginson et al., 2010), instead of standardized or validated instrument, thus weakening the robustness of validity results. Lastly, although the 12-item ZBI has been tested as having the best performance in previous studies that were mostly conducted in western countries (O'Rourke and Tuokko, 2003; Higginson et al., 2010; Hagell et al., 2017), it does not incorporate some cultural-sensitive salient concept in Asian societies (Lim et al., 2014), which may limit its use across countries that share similar Confusianism traditions (e.g., Japan, South Korea, and China) (Zhou et al., 2016; Lin et al., 2017b).

In light of the above-mentioned limitations of previous studies, the present study was conducted with the purpose of reassessing the psychometric properties of ten short forms of ZBI-22 among Chinese family caregivers of people with schizophrenia using a series of standardized or validated instrument for validity test.

## Materials and Methods

### Participants and Procedure

This is a descriptive, cross-sectional study with partial data coming from the research entitled “Family burden and caregiving experiences of schizophrenia in Chinese rural communities” (Yu et al., 2017), conducted in Ningxiang County, Hunan Province of China. The research recruited a sample of 352 primary caregiver s of schizophrenia through China's first program for treatment and management of serious mental illness (“Central Government Support for the Local Management and Treatment of Severe Mental Illnesses Project,” 686 Project) (Ma, 2012). The care recipient must be registered in the 686 Program and fulfilling the Chinese Classification of Mental Disorders-3(CCMD-3) or the International Classification of Diseases-10 (ICD-10) criteria for schizophrenia as diagnosed by special psychiatrist. The primary caregiver must be a family member who is living with the patient and taking the most responsibility of caring, 16 years old or above, and able to understand and communicate. After excluding 14 participants who refused to participate and 11 withdrawals, our final sample included 327 community-dwelling primary caregivers of patients with schizophrenia.

Data collection was conducted from October 2015 to January 2016. A team of nine post-graduates from the School of Public Health of Central South University were recruited as interviewers. The interviewers have a background of public health, psychology or psychiatry. All interviewers received a 1-week uniform formal training to conduct the interviews provided by a psychologist before the formal study. The training was composed of half lecturing and half practice of role plays. All subjects gave written informed consent in accordance with the Declaration of Helsinki. The protocol was approved by the Institutional Review Board of the Xiangya School of Public Health of Central South University. We paid door-to-door visit at the primary caregivers' home and conducted face-to-face interviews with them for an average duration of 50–70 min, after obtaining written consent from primary caregivers for the study. Details of the study enrollment and procedure have been published elsewhere (Yu et al., 2017).

### Instruments

#### Zarit Burden Interview (ZBI)

The ZBI-22 (Table 1) consists of 22 items scored in 5-point Likert scale from 0 (never) to 4 (nearly always), except for the final item on global burden, rated from 0 (not at all) to 4 (extremely). The total score ranges from 0 to 88 with higher scores indicating higher burden. In addition, several short forms of the ZBI-22 have been proposed as a rapid screening tool, which are scored according to the same principle as the original ZBI-22. These short forms include: the 14-item ZBI by Knight et al. (2000), two different 12-item ZBIs by Ballesteros et al. (2012) and Bedard et al. (2001), the 8-item ZBI by Arai et al. (2003), two different 7-item ZBIs by Zhou (2011) and Gort et al. (2005), the 6-item ZBI by Higginson et al. (2010), two different 4-item ZBIs by Gort et al. (2010) and Bedard et al. (2001), and finally, 1-item ZBI by Higginson et al. (2010) (Table 1). In the present study, the Chinese version of ZBI showed acceptable internal consistency with a McDonald's ω coefficient of 0.89.

#### Family Burden Interview Schedule (FBIS)

The FBIS (Pai and Kapur, 1981) was used to assess family burden and consists of 24 items rated on a 3-point Likert scale from 0 (no burden) to 2 (serious burden). The total scores range from 0 to 48 with higher scores showing higher burden. In the present study, the Chinese version of FBIS showed acceptable internal consistency with a McDonald's ω coefficient of 0.87.

#### Global Assessment of Functioning (GAF)

The GAF was used to assess patient function and consists of only one 100-point single item covering three major domains: social functioning, occupational functioning, and psychological functioning (American Psychiatric Association, 1994). The total scores range from 1 to 100, with higher scores indicating higher function. Examples are given for each ten-level interval.

#### Patient Health Questionnaire (PHQ-9)

The PHQ-9 (Spitzer et al., 1999) was used to assess caregivers' depression symptoms and consists of 9 items rated on a 4-point Likert scale from 0 (not at all) to 3 (nearly every day). The total scores range from 0 to 27, with a cut-off point of 10 differentiating depression and non-depression (Manea et al., 2015). The Chinese version of the PHQ-9 demonstrated good internal consistency in the current study with a McDonald's ω coefficient of 0.89.

#### Generalized Anxiety Disorder Scale (GAD-7)

The GAD-7 (Spitzer et al., 2006) was used to assess caregivers' anxiety and consists of 7 items rated on a 4-point Likert scale from 0 (not at all) to 3 (nearly every day). The total scores range from 0 to 21, with a cut-off point of 10 differentiating anxiety and non-anxiety (Schalet et al., 2014). The Chinese version of the GAD-7 demonstrated good internal consistency in the current study with a McDonald's ω coefficient of 0.91.

### Data Analysis

For the first step, internal consistency of all short scales was examined with McDonald's ω coefficient (McDonald, 1999; Zhang and Yuan, 2016). An ω value of 0.7–0.9 for long scales and 0.6 for short scales (e.g., four items) indicate good internal consistency, while an ω value of >0.9 suggests redundant items (Youden, 1950; Nunnally and Bernstein, 1994).

Secondly, validity of the scales was tested and compared for the following three tests: concurrent validity, known group's validity and criterion validity. We first tested the normal distribution of FBIS score, GAF score, and GAD score using sktest and found none of them fit normal distribution (*p* < 0.01), and thus used non-parametric testing in the following validity testing.

Concurrent validity was measured using Spearman correlations with expected significant negative correlations with patient's function (GAF score), as well as significant positive correlations with caregiver's depression (PHQ-9 score), and anxiety symptoms (GAD-7 score) (Ji et al., 2014; Wang et al., 2017; Sun et al., 2018; Yu et al., 2018). We further performed Fisher r-to-Z test to compare the statistical significance of different correlations.

Known group's validity was assessed using Mann-Whitney *U* tests. We expect that caregivers with physical disease exhibited higher caregiver burden than those without physical disease (Yu et al., 2017). Also, caregiver burden varies according to different caregiving roles, such as parents, spouse, and others (Chang et al., 2017; Yu et al., 2017).

Criterion-related validity were measured by Spearman correlations between short-form total scores and the gold standard—FBIS total scores, with an expected correlation coefficient of above 0.7 (Higginson et al., 2010). The coefficient of determination (*r*^2^) was also computed to assess how much the variance of the gold standard can be explained by respective short forms.

Thirdly, the discriminative performance of the short forms was assessed and compared with the receiver operating characteristic (ROC) curve (Coffin and Sukhatme, 1997), using the ZBI-22 cut-off 21 as the reference (Zarit et al., 1985a). The ROC was constructed by plotting sensitivity against 1-specificity, with each point representing a sensitivity/1-specificity pair corresponding to a particular cutoff value. The closer a ROC plot is to the upper-left corner, the higher the overall accuracy of the test. A point closest to (0, 1) on the ROC curve indicates the ideal condition (100% sensitivity and 100% specificity) (Coffin and Sukhatme, 1997). The areas under the curves (AUC) were calculated using the trapezoidal method (Weinstein et al., 1980; Coffin and Sukhatme, 1997) to represent the scale's the ability to correctly classify those with and without burden. The range of the AUC is 0.5–1.0. A discriminative test is considered perfect if AUC = 1.0, good if AUC = 0.8–1.0, moderate if AUC = 0.6–0.8, and poor if AUC = 0.5–0.6; an area of 0.5 reflects a random rating model (Weinstein et al., 1980). 95% Confidence intervals of AUCs were computed. A *P*-value below 0.05 was considered as statistically significant. Besides, cut-points for the ZBI short forms were estimated based on the Youden index (Fischer et al., 2003).

All analyses were carried out using SPSS 16.0 (SPSS Inc., Chicago, IL, USA) and R 3.5.1.

## Results

Table 2 shows the descriptive data on the sample. The mean (SD) age of the caregivers was 57.7 (12.5) years, and most of them were married (82.3%) and with primary education (59.9%). Slightly more than half of the caregivers were female (53.8%) and employed (52.9%). Most of their relationships to the care-recipient were parents (43.8%) and spouses (33.7%). The caregivers spent a median (q1–q3; minimal–maximum) of 15 years (9–25; 1–49) in caring for the patients. The median score of patient function as measured by GAF was 40.0 (20.0–61.0; 1–99). The mean score of depression, anxiety, and family burden as measured by PHQ-9, GAD-7, and FBIS were 9.75 (SD: 7.31), 9.31 (SD: 6.61), 23.62 (SD: 9.76), respectively.

**Table 2:** Characteristics of Family Caregivers (*n* = 327).

**Variables**		**Findings**
Age (years)	mean (SD; min–max)	57.7 (12.5; 17–81)
Gender	Male	151 (46.2)
	Female	176 (53.8)
Marriage	Married	269 (82.3)
	Others^*^	58 (17.7)
Occupation	Employed	173 (52.9)
	Unemployed	154 (47.1)
Education	Primary	196 (59.9)
	Middle	87 (26.6)
	High	44 (13.5)
Relationship with the patients	Parents	144 (43.8)
	Spouses	111 (33.7)
	Others^#^	72 (22.5)
Care duration (years)	md (q1–q3; min–max)	15 (9–25; 1–49)
Patient function (GAF^a^)	md (q1–q3; min–max)	40 (20–61; 1–99)
Depression (PHQ-9^b^)	mean (SD; min–max)	9.75 (7.31; 0–27)
Anxiety (GAD-7^c^)	mean (SD; min–max)	9.31 (6.61; 0–21)
Family burden (FBIS^d^)	mean (SD; min–max)	23.62 (9.76, 0–44)

* *Others include single, divorced, or widowed*.

# *Others include siblings, children, and cousins*.

a *GAF, Global Assessment of Functioning*.

b *PHQ-9, Patient Health Questionnaire-9*.

c *GAD-7, Generalized Anxiety Disorder Scale*.

d *FBIS, Family Burden Interview Schedule*.

Reliability and validity results were shown in Table 3. Reliability was generally good with McDonald's ω coefficient ranging from 0.69 to 0.85 across various ZBI formats, except for the Gort ZBI-4 (ω = 0.58). The low ω value for the Gort ZBI-4 may be explained by the property of the McDonald's ω, which is not an independent measure. McDonald's ω is sensitive to the number of items and increase with increased item numbers (Sijtsma, 2009). Based on this theory, we believe a ω of 0.58 being acceptable for such a small item scale like the Gort ZBI-4. Among all short forms, the Ballesteros ZBI-12 showed the highest ω of 0.85, followed closely by Bedard ZBI-12, the Arai ZBI-8, and Zhou ZBI-7 (ω = 0.84 for all).

**Table 3:** Reliability and Validity of the ZBI short forms.

**Property**	**Higginson ZBI-1 (Higginson et al., 2010)**	**Bedard ZBI-4 (Bedard et al., 2001)**	**Gort ZBI-4 (Gort et al., 2010)**	**Higginson ZBI-6 (Higginson et al., 2010)**	**Gort ZBI-7 (Gort et al., 2005)**	**Zhou ZBI-7(Zhou, 2011)**	**Arai ZBI-8 (Arai et al., 2003)**	**Bedard ZBI-12 (Bedard et al., 2001)**	**Ballesteros ZBI-12 (Ballesteros et al., 2012)**	**Knight ZBI-14 (Knight et al., 2000)**	**ZBI-22 (Zarit et al., 1985a)**
**INTERNAL CONSISTENCY**											
McDonald's ω	–	0.69 (0.67, 0.74)	0.58 (0.53, 0.65)	0.77 (0.73, 0.79)	0.80 (0.77, 0.82)	0.84 (0.82, 0.87)	0.84 (0.81, 0.85)	0.84 (0.84, 0.86)	0.85 (0.83, 0.87)	0.83 (0.78, 0.85)	0.89 (0.88, 0.90)
**CONCURRENT VALIDITY**^**&**^											
Patient function (GAF^*a*^)	−0.34^**^	−0.42^**^	−0.36^**^	−0.46^**^	−0.47^**^	−0.44^**^	−0.46^**^	−0.44^**^	−0.48^**^	−0.43^**^	−0.46^**^
Carer depression (PHQ-9 ^*b*^)	0.49^**^	0.57^**^	0.56^**^	0.63^**^	0.65^**^	0.63^**^	0.61^**^	0.60^**^	0.65^**^	0.58^**^	0.65^**^
Carer anxiety (GAD-7 ^*c*^)	0.45^**^	0.51^**^	0.55^**^	0.55^**^	0.56^**^	0.55^**^	0.53^**^	0.51^**^	0.58^**^	0.50^**^	0.56^**^
**KNOWN GROUPS' VALIDITY**											
Carers with disease, md (q1–q3)	4 (3, 4)	8 (5, 12)	8 (5, 12)	12 (6, 16)	15 (9, 20)	16 (9, 21)	15 (8, 21)	22 (12, 30)	27 (18.33)	26 (16, 34)	46 (32, 56)
Carers without disease, md (q1–q3)	3 (2, 4)	7.5 (2, 12)	7.5 (4, 10)	8 (3, 16)	11 (6, 20)	12.5 (6, 20)	12 (5, 23)	18 (9, 29)	21 (11, 32)	21 (12, 34)	37.5 (20.75, 56.5)
**d**_**cohen**_ **(95% CI)**	**0.412 (0.167, 0.657)**	0.226 (−0.018, 0.469)	**0.284 (0.04, 0.528)**	**0.298 (0.053, 0.542)**	**0.325 (0.080, 0.569)**	0.275 (0.03, 0.518)	0.183 (−0.061, 0.427)	0.223 (−0.02, 0.467)	**0.385 (0.140, 0.630)**	0.230 (−0.014, 0.474)	**0.297 (0.051, 0.543)**
**P**	**0.008**	0.142	**0.045**	**0.033**	**0.027**	0.066	0.226	0.087	**0.005**	0.089	**0.043**
1. Carers being Parents, md (q1–q3)	4 (3, 4)	9 (5, 12)	9 (5, 12)	12.5 (6, 16)	16 (9, 20)	16 (11, 23)	16 (9, 20)	23 (15, 32)	29 (20, 34)	27 (18, 35)	49 (36.25, 59)
2. Carers being Spouse, md (q1–q3)	3 (2, 4)	8 (4, 11)	8 (5, 11)	10 (6, 15)	13 (8, 18)	13 (7, 19)	13 (8, 18)	20 (11, 28)	23 (13.75, 32)	23 (15, 32)	41 (29.5, 52)
3. Carers being others, md (q1–q3)	3 (2, 4)	8 (4, 10)	6 (3, 9)	8 (3, 15)	11 (6, 20)	8 (3, 15)	11.5 (6, 21)	18 (10, 27)	21 (11, 32)	18.5 (12, 30)	35 (20.75, 54.25)
**Eta-squared (95% CI)**	**0.080 (0.028, 0.140)**	0.013 (0.046)	**0.029 (0.001, 0.071)**	**0.025 (0.00, 0.065)**	**0.035 (0.003, 0.081)**	**0.034 (0.003, 0.080)**	**0.041 (0.006, 0.089)**	0.017 (0.054)	**0.046 (0.008, 0.097)**	0.020 (0.058)	**0.038 (0.005, 0.086)**
**P**^**#**^	**<0.001**	0.141	**0.002**	**0.022**	**0.008**	**0.010**	**0.002**	0.051	**0.001**	**0.021**	**0.002**
Group comparison	**1 > 2, 3**	–	1, 2 > 3	1 > 3	**1 > 2, 3**	**1 > 2, 3**	**1 > 2, 3**	–	**1 > 2, 3**	1 > 3	**1 > 2, 3**
**CRITERION-RELATED VALIDITY**^**&**^											
FBIS^d^ correlation	0.57^**^	0.67^**^	0.67^**^	0.73^**^	0.75^**^	0.71^**^	0.67^**^	0.70^**^	0.74^**^	0.70^**^	–
FBIS^d^ coefficient of determination (r2)	0.34	0.45	0.44	0.53	0.56	0.50	0.45	0.49	0.55	0.49	–

*Bold values means significant at p < 0.05 or confidence intervals not straddling 0*.

** *P < 0.01*.

& *Spearman correlations*.

# *after Bonferroni correction*.

a *GAF, Global Assessment of Functioning*.

b *PHQ-9, Patient Health Questionnaire-9*.

c *GAD-7, Generalized Anxiety Disorder Scale*.

d *FBIS, Family Burden Interview Schedule*.

External concurrent validity was in general accordance with expectations and similar across the various ZBI forms, with significant negative correlations with GAF score (*r* = −0.34 to −0.48), and significant positive correlations with PHQ-9 score (*r* = 0.49–0.65) and GAD-7 score (*r* = 0.45–0.58). Among all the various ZBI forms, the Ballesteros ZBI-12 showed the highest correlations with the scores of GAF, PHQ-9, and GAD-7. A subsequent Fisher r-to-Z test found no statistical differences among those correlations of various short forms, indicating that all short forms performed equally well in concurrent validity.

For known groups' validity, among all short forms, five showed significantly higher burden scores in caregivers with disease than those without disease. For caregiving role and caregiver burden, eight forms exhibited significant higher burden scores in parent caregiver group, yet only five showed exactly the same pattern as the full ZBI scale: parents > spouse, others. Among all short forms, only three displayed favorable known group validity consistently: Higginson ZBI-1, the Gort ZBI-7, and the Ballesteros ZBI-12.

For criterion-related validity, all ZBI short forms showed significant high correlations with the FBIS criterion (*r* = 0.67–0.75) except for Higginson ZBI-1 (r = 0.57), which may be explained by the only one item in Higginson ZBI-1. Among all short forms, Gort ZBI-7 showed the highest criterion-related validity (*r* = 0.75), followed closely by Ballesteros ZBI-12 (*r* = 0.74) and Higginson ZBI-6 (*r* = 0.73).

**Figure 1:**
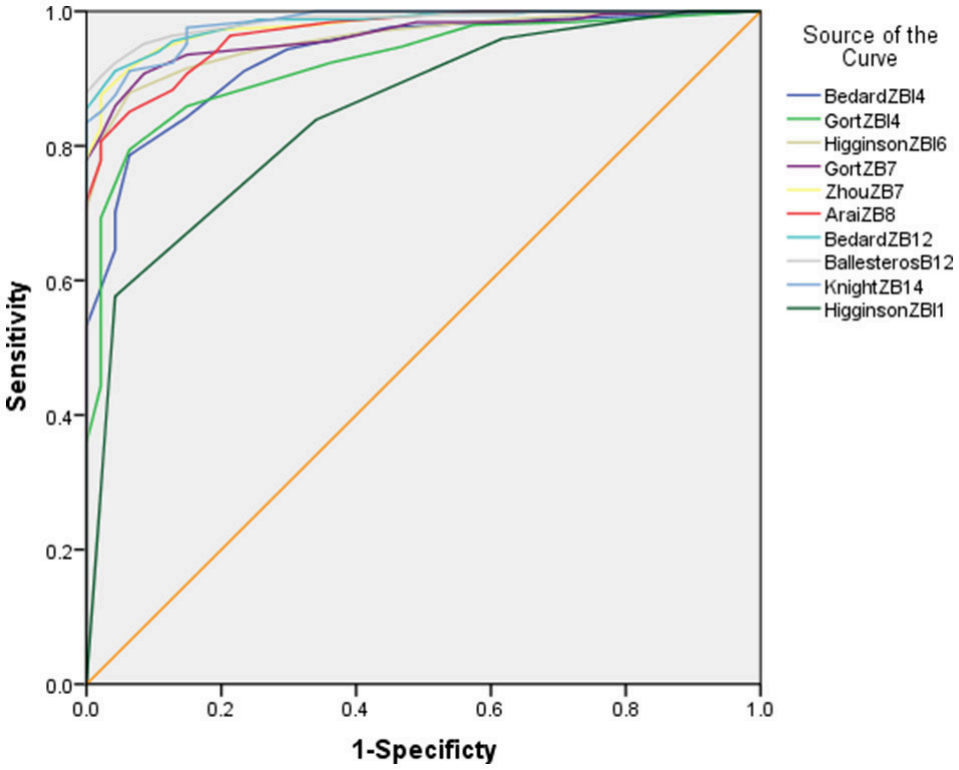
Receiver operating characteristic curves for various short-form versions of the Zarit Burden Interview (ZBI) and areas under the curve (AUC, 95% confidence interval [CI]). A total score of 21 on the full-scale ZBI as the cutoff value between low and high burden.

Optimal cut-points according to the Youden index relative to the gold standard cut-point of 21 on the ZBI-22 are shown in Figure 1 and Table 4. All shorter versions were overall successful in differentiating low- and high-burden individuals with AUCs ranging from 0.85 to 0.99. Among all short forms, the Ballesteros ZBI-12 showed the highest AUC (0.99), followed closely by Knight ZBI-14, Bedard ZBI-12, and Zhou ZBI-7, all with the same AUC of 0.98.

**Table 4:** Cutpoint Estimations and ROC Curve Analyses of ZBI Short Forms Relative to the Suggested ZBI-22 Cutpoint of 21.

	**Cutpoint**	**Sensitivity**	**Specificity**	**Youden index (SE)**	**AUC**	***P***
Higginson ZBI-1 (Higginson et al., 2010)	4	0.58	0.96	0.53	0.85 (0.79, 0.91)	< 0.001
Bedard ZBI-4 (Bedard et al., 2001)	6	0.72	0.94	0.72	0.93 (0.90, 0.97)	< 0.001
Gort ZBI-4 (Gort et al., 2010)	6	0.79	0.94	0.73	0.92 (0.89, 0.96)	< 0.001
Higginson ZBI-6 (Higginson et al., 2010)	6	0.88	0.94	0.82	0.96 (0.94, 0.98)	< 0.001
Gort ZBI-7 (Gort et al., 2005)	8	0.91	0.92	0.82	0.96 (0.94, 0.98)	< 0.001
Zhou ZBI-7 (Zhou, 2011)	9	0.88	0.98	0.85	0.98 (0.97, 0.99)	< 0.001
Arai ZBI-8 (Arai et al., 2003)	8	0.85	0.94	0.79	0.97 (0.95, 0.99)	< 0.001
Bedard ZBI-12 (Bedard et al., 2001)	12	0.88	0.98	0.86	0.98 (0.97, 0.99)	< 0.001
Ballesteros ZBI-12 (Ballesteros et al., 2012)	14	0.90	0.98	0.88	0.99 (0.98, 1.00)	< 0.001
Knight ZBI-14 (Knight et al., 2000)	17	0.84	1.00	0.84	0.98 (0.97, 0.99)	< 0.001

## Discussion

This study assessed and validated ten short forms of the Zarit Burden Interview (ZBI) among family caregivers of people with schizophrenia. We found general support for the psychometric testing of all ten short forms. While previous studies proposed either the Bedard 12-item ZBI (Higginson et al., 2010; Hagell et al., 2017) or the Ballesteros 12-item ZBI (Lin et al., 2017b) as the best one, our comparison produced two structures that outperformed other structures in comprehensive psychometric properties: the Ballesteros 12-item and the Gort ZBI-7.

Compared to the full ZBI form, the short forms are easier to administer and less time-consuming due to the abridged items. The use of ZBI short forms facilitates rapid identification of caregiver burden and quick further assessment or referral in busy clinical situations and is thus favored by physicians. Also, it alleviates respondent burden of caregivers who focus more on the patients they cared for than their own feelings and thus only want to answer the briefest questionnaire.

Four key findings emerge from our analysis. First, we found high internal consistency of all short versions of the ZBI with McDonald's ω ≥ 0.70, except for Gort ZBI-4 (McDonald's ω = 0.58). On the one hand, it reflects the property of the McDonald's omega coefficient that is sensitive to the number of items and decreases with reduced items (Sijtsma, 2009). On the other hand, it may imply the potential item redundancy of the full ZBI scale and propose better applicability of short forms, as shown in another study (Lin et al., 2017b).

Second, while all ten ZBI short forms uniformly exhibited desirable concurrent validity correlations and criterion-related validity, there is inconsistency in the result of known groups' validity. Although caregiver burden has been widely proven to be positively associated with physical disease in the literature (Sethabouppha and Kane, 2005; Gater et al., 2014; Yu et al., 2017), only five ZBI short forms demonstrated significant higher burden scores in caregivers with disease than those without in the current study. Moreover, past studies have shown that caregiver burden varies according to caregiving role, with the majority supporting the highest burden in parents, followed by spouse, siblings, children, and others in turn (Lu, 2009; Hsiao and Tsai, 2014a,b). In the present study, eight out of ten short forms have successfully distinguished caregiver burden among various caregiving roles, while only 5 short forms showed exactly the same pattern as the full ZBI scale: parents > spouse, others. In sum, only the Higginson ZBI-1, the Gort ZBI-7, and the Ballesteros 12-item ZBI displayed favorable known group validity consistently, which were highly recommended for future use in screening for various groups.

Third, our findings found the Ballesteros 12-item and the Gort ZBI-7 outperformed other short forms with almost equally good performance in psychometric testing. This finding partly supports Lin's (Lin et al., 2017b) previous conclusion of Ballesteros 12-item ZBI as the best one, yet conflicts with the findings of Hagell and Higginson, both proposed the Bedard 12-item ZBI (Higginson et al., 2010; Hagell et al., 2017) as the best one. A careful comparison between the Gort ZBI-7 and the Bedard ZBI-12 showed that the latter included all items of the former except for the item 22 “Overall, how burdened do you feel in caring.” One possible explanation may be that item 22 acts as an overall summary of the whole scale and is more representative of the full ZBI score than any other item. In fact, some authors even propose reducing the whole scale into an extreme one item—the Higginson ZBI-1 as a rapid assessment of caregiver burden (Higginson et al., 2010; Ballesteros et al., 2012). However, the measurement of item 22 alone is not enough to represent the whole scale because it is based on the answering and learning process of the all previous items, leading to its analysis in isolation from the rest of the information unpractical (Ballesteros et al., 2012). Another explanation may be that the item 22 acts as more like a supplement to the previous items than a summary, as shown in our analysis, the Gort ZBI-7 is actually a combination of the Higginson ZBI-1 and Higginson ZBI-6 but performed much better than the other two. It is likely that item 22 may have measures some salient concept that other items may not be able to capture, especially in Asian cultures. As a result, this item is an integral part of the scale that can neither be isolated, nor deleted.

Our fourth finding is cut-off values ranging from 4 to 17 for the various short forms of ZBI relative to that of the ZBI-22. The results were slightly different from those previously identified among family caregivers of people with cancer, brain injury, and dementia (Higginson et al., 2010; Hagell et al., 2017), which may be explained by the various sample as mentioned above and provides useful guidance for future use of short forms among such a population. Furthermore, all short forms displayed good AUC values of above 0.8, further corroborating their high discriminative performances to distinguish those with and without burden, which accords with past studies (Higginson et al., 2010; Hagell et al., 2017).

The study falls short in several aspects. First, this is a cross-sectional study with no information on short forms' responsiveness to change, which is important for psychometric property in intervention and longitudinal studies (Kirshner and Guyatt, 1985). Second, we did not run test-retest reliability in the current study. Further study may consider collecting data in multiple time points to detect responsiveness to change and test-retest reliability. Third, we did not test the factorial structure of the ZBI and treated all the different short forms of ZBI as a unidimensional structure. Conflicting evidence exists on the factor analysis of both the 22-item ZBI and its short forms in past studies (Ballesteros et al., 2012; Branger et al., 2016; Tang et al., 2017). One possible explanation may be that factor analysis is sample dependent and may vary according to different study populations, thus making testing and comparing factor structures among various short forms in the current single study population implausible (Yu et al., 2015), future research may benefit from adding factor analysis into comparison among short forms across diverse populations. Fourth, the finding of the current study is based on quantitative analysis of different short form versions, there is a lack of qualitative evaluation of each item for cultural adaptation, future research may consider mixed-method comparison for these short forms. Finally, the sample came from one single rural county of Hunan province, which may limit the study's representativeness. Future multi-center large sample comparative studies are warranted for firmer conclusions.

In conclusion, our observations provide initial support for the psychometric properties of various ZBI short forms for use among family caregivers of people with schizophrenia. The Ballesteros ZBI-12 and the Gort ZBI-7 showed the best performance in reliability and validity, which paves the way for future studies to further utilize and validate the scales.

## Data Availability Statement

The raw data supporting the conclusions of this manuscript will be made available by the authors, without undue reservation, to any qualified researcher.

## Author Contributions

YY and SX contributed to the conception and design of the study. YY, ZL, WZ, XC, MH, and XZ contributed to the research conduction and data collection. YY and ZL contributed to data analyses and interpretation, analysis tools or data. YY drafted the article while ZL, WZ, XC, MH, XZ, and SX critically appraised it and revised it. All authors approved the final version of manuscript for submission and publication.

### Conflict of Interest Statement

The authors declare that the research was conducted in the absence of any commercial or financial relationships that could be construed as a potential conflict of interest.
